# The Ethanol Extract of *Osmanthus fragrans* Flowers Reduces Oxidative Stress and Allergic Airway Inflammation in an Animal Model

**DOI:** 10.1155/2013/304290

**Published:** 2013-12-10

**Authors:** Chien-Ya Hung, Fu-Long Huang, Li-Shian Shi, Shuk-Man Ka, Jing-Yao Wang, Yu-Cheng Tsai, Tsung-Jen Hung, Yi-Ling Ye

**Affiliations:** ^1^Department of Food Nutrition, Chung Hwa University of Medical Technology, Tainan 71703, Taiwan; ^2^Graduate Institute of Food Science, National Chiayi University, Chiayi 60004, Taiwan; ^3^Department of Biotechnology, National Formosa University, Yunlin 63201, Taiwan; ^4^Graduate Institute of Aerospace and Undersea Medicine, National Defense Medical Center, Taipei 11490, Taiwan; ^5^Department of Graduate Institute of Biomedical Science, Chung Hwa University of Medical Technology, Tainan 71703, Taiwan

## Abstract

The *Osmanthus fragrans* flower, a popular herb in Eastern countries, contains several antioxidant compounds. *Ben Cao Gang Mu*, traditional Chinese medical literature, describes the usefulness of these flowers for phlegm and stasis reduction, arrest of dysentery with blood in the bowel, and stomachache and diarrhea treatment. However, modern evidence regarding the therapeutic efficacy of these flowers is limited. This study was aimed at assessing the antioxidative effects of the ethanol extract of *O. fragrans* flowers (OFE) *in vivo* and evaluating its antioxidant maintenance and therapeutic effect on an allergic airway inflammation in mice. After OFE's oral administration to mice, the values obtained in the oxygen radical absorbance capacity assay as well as the glutathione concentration in the lungs and spleens of mice increased while thiobarbituric acid reactive substances decreased significantly, indicating OFE's significant *in vivo* antioxidant activity. OFE was also therapeutically efficacious in a mouse model of ovalbumin-induced allergic airway inflammation. Orally administered OFE suppressed ovalbumin-specific IgE production and inflammatory cell infiltration in the lung. Moreover, the antioxidative state of the mice improved. Thus, our findings confirm the ability of the *O. fragrans* flowers to reduce phlegm and suggest that OFE may be useful as an antiallergic agent.

## 1. Introduction

*Osmanthus fragrans*, known as sweet olive, tea olive, and fragrant olive, is a species of Oleaceae native to southwestern China [1]. It is widely cultivated as an ornamental plant for its fragrant flowers in Taiwan, southern Japan, southern China, Europe, North America, and elsewhere. The flower of *O. fragrans*, called *Kwai-fah* in China, has been used as a beverage and as an additive for tea and foods such as cake, pastry, paste, vinegar, and liqueurs. It is popular because of its delicate fruity/floral aroma. Various volatile components of the flowers are also used, primarily for perfumes, flavors, and aromatherapy. It was recorded in *Ben Cao Gang Mu* that the *O. fragrans *flower was used to reduce phlegm and stasis as well as to arrest dysentery with blood in the bowel. Traditional Chinese medicine has also suggested the use of *O. fragrans *to treat weakened vision, halitosis, panting, asthma, cough, toothache, stomachache, diarrhea, and hepatitis. However, modern evidence for the biomedical use of the ethanol extract of *O. fragrans *flowers (OFE) is limited.

Overproduction of free radicals can cause oxidative damage and may eventually lead to chronic diseases. Many plants contain free radical-scavenging molecules such as phenolic acids and flavonoids, which show strong antioxidant activity. There are some reports of antioxidant activity of *O. fragrans. *Lee et al. demonstrated that the dried flowers of *O. fragrans *contain abundant phenols and flavonoids and exhibit antioxidant activity in the ferric reducing ability of plasma (FRAP) assay, 1,1-diphenyl-2-picryl-hydrazyl (DPPH) radical-scavenging activity, and hydroxyl anion scavenging ability [2]. Our group has also analyzed many antioxidant components of OFE and found that the antioxidant activityof* O. fragrans* is slightly weaker than that of green tea [3]. In the aforementioned study, five phenolic compounds, tyrosyl acetate (**1**), (+)-phillygenin (**2**), (8*E*)-ligustroside (**3**), rutin (**4**), and verbascoside (**5**), were isolated from the CHCl_3_ layer of *O. fragrans*. Evaluation of the antioxidative properties of the isolated compounds **2**, **4**, and **5** revealed strong DPPH radical-scavenging activity, with IC_50_ values of 19.1, 10.3, and 6.2 *μ*M, respectively. These isolates also exhibited an H_2_O_2_-scavenging ability, with IC_50_ values of 10.5, 23.4, and 13.4 *μ*M, respectively.

Although modern evidence for the *in vivo *therapeutic activity of *O. fragrans *is yet to be provided, a few studies have examined the *in vitro *bioactivity of OFE. For example, OFE inhibited lipid peroxidation occurring through ferrous chloride in the mitochondria in rat brain, liver, heart, and kidney [2]. OFE may also exert neuroprotective actions through the upregulation of the AKT survival pathway, which attenuates neurotoxicity [2]. Several lignans isolated from the flowers of *O. fragrans *var.* aurantiacus* inhibited nitric oxide production in lipopolysaccharide (LPS)-stimulated RAW 264.7 macrophages [4].

Asthma is a chronic respiratory disease characterized by airway inflammation and airway hyperresponsiveness (AHR). Previous reviews have highlighted the importance of reactive oxygen/nitrogen species in airway inflammation [5, 6]. Reactive oxygen species (ROS) also contribute to lipid peroxidation. The pathologic effects of lipid peroxidation, including epithelial cell activation, disruption, or death, smooth muscle contraction, airway hypersensitivity, and increased mucus secretion, are all observed in asthma [7]. Losing control of intracellular oxidants to environmental cellular stress also may lead to allergic disorders [8]. For example, the polarization of the helper T cells Th1/Th2 balance is dependent on the intracellular thiol-redox status of macrophages [9]. In another study, Pazmandi et al. observed that H_2_O_2_-exposed plasmacytoid dendritic cells (pDCs) provided stronger stimuli for Th2 than for Th1 differentiation upon autologous activation (cocultivation with naïve autologous T cells) when compared to untreated pDCs [10]. Furthermore, enhanced oxidative stress may contribute to the progression or perpetuation of existing airway inflammation through enhanced AHR [11, 12] via airway smooth muscle contraction [13], mucus hypersecretion [14], epithelial shedding [15], vascular exudation [16, 17], and induction of various proinflammatory chemical mediators because of accumulation of inflammatory cells in the lower respiratory tract [18]. All of these actions are believed to be related to severe asthma [19–21].

The increase in ROS during an asthma exacerbation might overwhelm endogenous antioxidant defenses. Glutathione (GSH) is a key antioxidant in the lining fluid of the respiratory tract. Disturbed GSH status is reported in asthma for total [22] and for oxidized [23] GSH. Total GSH concentration, which includes the oxidized form GSSG, is higher in the bronchial and alveolar fluid in patients with mild asthma [22]. A recent report suggested that increasing GSH levels and the GSH/GSSG ratio with *γ*-glutamylcysteine ethyl ester (*γ*-GCE) ameliorated bronchial asthma by altering the Th1/Th2 cell imbalance through interleukin (IL-) 12 production from antigen presenting cells (APC) and by suppressing chemokine production and eosinophil migration [24]. Enhancing intracellular GSH can also decrease the release of cytokines from lung cells by decreasing NF-*κ*B activation [25, 26]. In addition, GSH has recently been reported to attenuate IL-13 induced asthma in mice [27].

Liao et al. investigated the total antioxidant capability in serum and the activity of the antioxidant enzymes superoxide dismutase (SOD) and glutathione peroxidase in 46 asthmatic children and 52 normal controls [28]. The total antioxidant capability in the serum of asthmatic children is significantly less than that found in the serum of controls. Plasma levels of lipid peroxides are also significantly increased in patients with asthma [29, 30].

The concentration of ascorbic acid and *α*-tocopherol in lung lining fluid is reportedly low in patients with mild asthma [23, 31]. Low dietary intake of vitamin C and manganese was found to be associated with an increased risk of bronchial activity [30]. Dietary supplements of vitamin C and vitamin E to asthmatics decrease the severity of pollutant-induced bronchial responsiveness [32]. Chang and Crapo [33] found that intratracheal treatment of ovalbumin (OVA-) challenged mice with the catalytic antioxidant, manganese (III) meso-tetrakis-(*N*-methylpyridinium-2-yl) porphyrin, having high SOD activity drastically reduced the severity of airway inflammation as evidenced by the reduced numbers of eosinophils, neutrophils, and lymphocytes found in bronchoalveolar lavage fluid.

The aim of the current study was to examine the mechanism of the preventive effect of OFE on allergic airway inflammation. The study comprised two parts: the first was to evaluate the antioxidation state following OFE administration and the second was to examine the immunomodulatory effects of OFE. We evaluated the antioxidative effects of OFE in naïve mice using the following methods: assessing the total antioxidant capacity in the lung and spleen by using the values obtained in the oxygen radical absorbance capacity (ORAC) assay, assessing the free radical-scavenging effects on DPPH, monitoring lipid peroxidation by measuring thiobarbituric acid reactive substances (TBARS), and determining the concentration of GSH. We used a mouse model of allergic airway inflammation to investigate the antiallergic and antioxidant effects of OFE as well as to determine organ weights.

## 2. Materials and Methods

### 2.1. Plant Material

Dried flowers of *O. fragrans* were collected from Nanto, Taiwan, in 2005-2006. They were examined and authenticated by Professor C. S. Kuoh, Department of Biology, National Cheng Kung University. A voucher specimen (no. Hung-0201) was deposited in the Herbarium of Chung Hwa University of Medical Technology.

### 2.2. Preparation of OFE

The dried flowers of *O. fragrans *were ground into a fine powder using a mill (RT-08, Rong Tsong, Taiwan), collected and sealed in a polyethylene plastic bag, and then stored at 0–4°C for further use. *O. fragrans *flowers (200 g) were soaked (72 h) in 75% ethanol (3 L) twice and filtered through Whatman no. 1 filter paper. The combined extracts were concentrated under reduced pressure and freeze-dried to provide a dark syrup that was stored at −20°C for further use. The extract was endotoxin free (≤0.1 E.U.) according to the Limulus amebocyte lysate (LAL) assay (Cambrex, Walkersville, MD).

#### 2.2.1. Determination of Total Phenolic Content in OFE

Following the method described by Yen and Hung [34], the sample solution in methanol (0.1 mL, 1 mg/mL) was well mixed with 2% Na_2_CO_3_ (2 mL). After 3 min, 50% Folin-Ciocalteu agent (0.1 mL) was added. The mixture was allowed to stand at room temperature (RT) for 30 min with intermittent mixing. The absorbance at 750 nm was recorded. A standard curve using gallic acid was prepared. The total phenolic content was expressed as gallic acid equivalents (mg of GAE per g extract).

#### 2.2.2. Determination of Total Flavonoid Content in OFE

Following the methods described by Woisky and Salatino [35] and also by Chang et al. [36], the sample solution (0.5 mL) was mixed with 95% EtOH (1.5 mL), 10% AlCl_3_ (0.1 mL), 1 M KOAc (0.1 mL), and distilled water (2.8 mL). The mixture was allowed to stand at RT for 30 min, and the absorbance was measured at 415 nm. The amount of sample solution was substituted by the same amount of a quercetin solution (0–200 *μ*g/mL) as a standard. The amount of 10% aluminum chloride was substituted by the same amount of distilled water to serve as a blank. The total flavonoid content was calculated from the plot of absorbance against quercetin concentration using linear regression analysis and expressed as quercetin equivalents (*μ*g of QE per g extract).

### 2.3. DPPH Free Radical-Scavenging Assay

DPPH is a stable free radical with a purple color that is reduced by antioxidants to a colorless compound. We employed DPPH in an assay modified from the method of Shimada et al. [37]. MeOH (3.8 mL), sample solution in methanol (0.2 mL, 1 mg/mL), and 1 mM DPPH solution (1.0 mL) were mixed well and left to stand in the dark at RT for 30 min. The final concentration of the sample was 40 *μ*g/mL. The absorbance at 517 nm was measured. The sample in methanol was used as a blank, while DPPH radical in methanol solution was used as a control. The DPPH radical scavenging activity was calculated according to the following equation:

(1) %  of  DPPH  radical  scavenging  activity =[1−(Asample−Ablank)Acontrol]×100,

where *A* is the absorbance at 517 nm.

The concentration providing 50% inhibition (IC_50_) of the DPPH radical-scavenging activity was calculated from the plot of the percent inhibition against sample concentration by using a linear regression analysis.

### 2.4. Animals

Female BALB/c mice between six and eight weeks of age were purchased from the National Laboratory Animal Center in Taiwan. The animal room was kept on a 12 h light : dark cycle and maintained at a constant temperature (25°C ± 2°C) and humidity. Animal care and handling conformed to the *NIH Guide for the Care and Use of Laboratory Animals*. All experiments were performed under protocols approved by the biotechnology department of National Formosa University's affidavit of approval of animal use protocol. Pentobarbital (intraperitoneal, i.p.) injections were used to anesthetize (10 mg/mL, 60 *μ*L per mouse) or sacrifice (10 mg/mL, 200 *μ*L per mouse) the mice.

For the results obtained in the antioxidant evaluation assays, mice were divided into two groups comprised of four BALB/c mice each. Group 1 (control) received only distilled water. Group 2 (OFE) received 1000 mg/kg body weight OFE daily for 14 days by oral gavage.

### 2.5. Organ Collection, Preparation, and Protein Quantization

After sacrifice, organs from the mice were collected and weighed. The spleens and lungs (1 g) were homogenized in PBS (pH 7.2) on ice by using a homogenizer (Motor Drives, Glas-Col Inc., Terre Haute, IN). The homogenate was then centrifuged at 2200 ×g for 10 min, and the filtrate was collected. Protein was determined by using the BCA assay kit (Pierce, Rockford, IL) using bovine serum albumin as a standard.

### 2.6. Determination of Antioxidant Activity

#### 2.6.1. Oxygen Radical Absorbance Capacity Assay (ORAC)

The total antioxidant activity of the organ samples was measured by using the oxygen radical absorbance capacity (ORAC) assay according to Chung et al. [38]. This assay was carried out in black-walled, 96-well plates at 37°C. All solutions were prepared in 75 mM phosphate buffer (Na_2_HPO_4_ : NaH_2_PO_4_, pH 7.0) and preincubated at 37°C for 30 min before use. Fifteen *μ*L of organ homogenate (diluted 100 times) and 100 *μ*L of 0.1 *μ*M *β*-PE (*β*-phycoerythrin) were transferred directly into the well to incubate for 10 min using the FLUOstar OPTIMA microplate reader system (Galaxy BMG LABTECH Inc., Cary, NC). We rapidly added 75 mM 2,2′-azobis (2-amidinopropane) dihydrochloride (AAPH, 85 *μ*L) and immediately measured the resulting fluorescence by using fluorescence filters with an excitation wavelength of 480 nm and an emission wavelength of 520 nm. The fluorescence was recorded at 5 min intervals for 120 min until the final value was less than 5% of the initial value. ORAC values from samples were calculated by using the following equation and expressed as Trolox equivalents: ORAC value (mM) = 20 × *k* × (*S*_sample_ − *S*_blank_)/(*S*_Trolox_ − *S*_blank_), where *k* was the sample dilution factor. The area under the curve (*S*) was calculated by the following equation:

(2) S=(0.5  +f5f0  +f10f0  +f15f0  +f20f0  +f25f0+⋯+f120f0)×5,

where *f*_0_ was the initial fluorescence reading at 0 min and *f*_*n*_ represented the measurement at time *n*.

#### 2.6.2. TBARS Assay

Lipid peroxidation was measured by determining the formation of malondialdehyde based on the presence of TBARS in the lung and spleen [39]. A standard curve was prepared using 1,1,3,3-tetraethoxypropane (Sigma, St. Louis, Mo). The organ filtrate (1 mL) and standard were mixed with 10% trichloroacetic acid (1 mL) 0.4% thiobarbituric acid (1 mL) and 0.2% butylated hydroxy toluene (BHT) (0.1 mL) (both reagents from Sigma, St. Louis, Mo). The reaction mixture was incubated at 95°C for 1 h, cooled under light-protected conditions, and then centrifuged at 2200 ×g for 10 min. The absorbance of the supernatant was measured using a microplate fluorescence reader (F-2500, Hitachi, Japan) in 96-well format with the excitation and emission filters set at 515 nm and 550 nm, respectively.

#### 2.6.3. Measurement of Glutathione

The glutathione concentration was determined according to the method described by Sedlak and Lindsay [40] with little modification. The organ homogenate (150 *μ*L) was mixed with 5% TCA solution (450 *μ*L). The resulting solution was centrifuged at 16770 ×g for 10 min to remove protein. The supernatant (30 *μ*L) and 0.01 M 5,5′-dithio-bis-(2-nitrobenzoic acid) (DTNB, 10 *μ*L) were added to 0.4 M Tris buffer (140 *μ*L, pH 8.9). The solution remained at RT for 5 min. The absorbance at 412 nm was measured by n ELISA Reader (VersaMax, Molecular Devices Inc., Sunnyvale, CA). A GSH solution (0–12 *μ*M) was used as standard. Tris buffer was used as blank. The plasma GSH content was calculated from the plot of absorbance against GSH concentration.

### 2.7. Establishment of the Animal Model of Allergic Airway Inflammation

**Figure 1:**
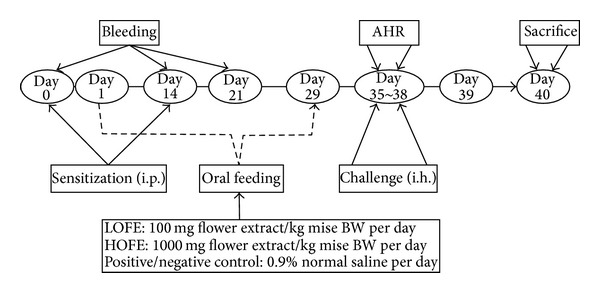
The schedule for the development of the OVA animal model of allergic airway inflammation and OFE administration. i.p.: intraperitoneal injection; i.h.: inhalation with OVA.

The schedule for OVA sensitization and challenge as well as the oral administration schedule of OFE are summarized in Figure 1. Briefly, the mice were sensitized with i.p. injections of OVA (20 *μ*g in 1x PBS; Sigma) mixed with alum (2 mg; Pierce, Rockford, III) as an adjuvant on day 0. On day 14, the mice were boosted with OVA (50 *μ*g) mixed with alum (2 mg). The mice that were given i.p. injections of 1x PBS served as negative controls. Either one of two doses of OFE in normal saline (200 *μ*L) (high OFE, 1000 mg/kg body weight or low OFE, 100 mg/kg body weight) or normal saline (for positive and negative control groups) was orally administered daily from day 1 to day 29. Each group consisted of six mice. On days 35–38, the mice were exposed to aerosolized 5% OVA for a 20 min period by placing them in a chamber. The aerosols were generated and conducted into the chamber using an ultrasonic nebulizer (DeVibiss, PA). The output of the nebulizer was 0.3 mL/min and the particles size produced was 0.5–5 *μ*m.

#### 2.7.1. Serum Levels of Anti-OVA Antibodies

After all groups of mice were anesthetized, they were bled from the retro-orbital venous plexus. Serum was collected after centrifugation at 12,000 ×g for 10 min. Sera anti-OVA IgE and IgG2a antibody titers were determined by ELISA. Briefly, 96-well microtiter plates were coated with OVA (10 *μ*g/mL). Serum samples were added to each well for an overnight period. Then biotin-conjugated anti-mouse IgE or IgG2a was added for 1 h at 37°C. Streptavidin-conjugated alkaline phosphatase was added for an additional 2 h at RT. Finally, the reaction was developed by 2,2′-azino-bis (3-ethylbenz-thiazoline-6-sulfonic acid), and the absorbance was determined at 420 nm in a microplate reader.

#### 2.7.2. Bronchoalveolar Lavage and Cell Differential Counts

After all groups of mice were anesthetized, they were bled from the retroorbital venous plexus and then sacrificed. Using a cannula, their lungs were immediately lavaged through the trachea three times with 1x HBSS (1 mL) minus ionized calcium and magnesium. The lavaged fluid was centrifuged at 400 ×g for 10 min at 4°C. After washing, the cells were resuspended in 1x HBSS (1 mL) and the total cell counts were determined using a hemocytometer. Cytocentrifuged preparations were stained with Liu's stain for differential cell counts. Based on standard morphologic criteria, a minimum of 200 cells was counted and classified as monocytes, lymphocytes, neutrophils, or eosinophils.

#### 2.7.3. Histopathological Study of Lung Organs

To evaluate the effects of OFE treatment, the lungs were immediately removed after lavage and fixed in a solution of 3% v/v formalin (in 0.01 M phosphate buffer, pH 7.2). The tissues were subsequently embedded in paraffin, cut into 5-*μ*m-thick sections, stained with hematoxylin-eosin, and examined by light microscopy for histopathological changes.

### 2.8. Statistical Analysis

Data from the ORAC and the TBARS assay as well the GSH concentration and various treatments are presented as mean ± SD (standard deviation). The Student's t test was used for comparison between treatments. Differences between two treatment groups or a treatment group compared with a negative or positive control group were considered statistically significant at *P* values less than 0.05, 0.01, or 0.001.

## 3. Results and Discussion

### 3.1. The Total Phenolic and Flavonoid Content in OFE

The total phenolic content in OFE was 367.9 ± 13.4 mg GAE/g extract, while the total flavonoid content was 45.0 ± 2.0 *μ*g QE/g extract. Flavonoids have been reported to induce antiallergic and anti-inflammatory effects [41]. For example, flavonoids showed a strong inhibition of IL-4 and IL-13 production, histamine release, and CD40 ligand expression in basophils [42]. The antiasthmatic effect by phenylpropanoid glycosides such as verbascoside has also been reported [43].

### 3.2. Antioxidant Activity and Free Radical Scavenging Capacity of OFE as Determined in the ORAC and DPPH Assays

The total antioxidant capacity of ethanol extract of OFE as determined in the ORAC assay was 0.4 ± 0.0 mM Trolox equivalents. The DPPH IC_50_ was 8.4 *μ*g/mL, which was better than that of the methanol extract (12.8 *μ*g/mL) of *O. fragrans* flowers [3], but less than Trolox (4.9 *μ*g/mL). Green tea, which has been proven to have a strong scavenging effect against DPPH radicals, has been reported to have antiallergic activity [44]. We found that in addition to the other antioxidant phenolic compounds, verbascoside and rutin are two major compounds in the ethanol extract of OFE (unpublished data). The antiallergic effects of verbascoside [43] and rutin [45] have also been reported. Together, these results support our decision to evaluate the antiallergic effects of the ethanol extract of OFE.

### 3.3. The Effects of Orally Administered OFE on Total Antioxidant Capacity, Lipid Peroxidation, and GSH Concentration in Lung and Spleen of BALB/c Mice

The oxidation of lipids, nucleic acids, or proteins may be involved in the oxidative damage of biological molecules. Additionally, oxidation is believed to play a role in the development of inflammatory diseases. For this reason, the total antioxidant capacity (as measured in the ORAC assay), GSH concentration, and lipid peroxidation (as measured in the TBARS assay) in mouse lung and spleen were investigated. There are no previous reports on the *in vivo *antioxidant effects of OFE.

We first evaluated the antioxidant capacity of the OFE extract in mice after oral administration of OFE for 14 days. Lungs and spleens from the OFE group demonstrated significantly higher values in the ORAC assay and higher concentrations of GSH than those in the control group (normal saline) (Table 1). In contrast, values derived from the TBARS assay were significantly decreased in the OFE group compared with those in the control group. Taken together, these results indicate that oral administration of OFE promotes antioxidant capacity and reduces lipid peroxidation in the lung and spleen of mice.

The phenolic antioxidants in OFE include tyrosyl acetate, (+)-phillygenin, (8*E*)-ligustroside, rutin, and verbascoside [3]. The verbascoside obtained from OFE has been reported to protect cell lines from free radical-induced oxidative stress as measured by TBARS [46] and *β*-amyloid-induced cell injury by attenuating ROS production [46]. However, the *in vivo *evaluation of the antioxidant effects of these five compounds needs further study. Oxidative stress is an important factor that contributes to the pathologic development of asthma. Given our results for the antioxidant capacity of OFE, we next evaluated OFE for its preventive effects in an animal model of allergic airway inflammation.

### 3.4. OVA-Specific Serum Antibody Levels in an Animal Model of Allergic Airway Inflammation

**Figure 2:**
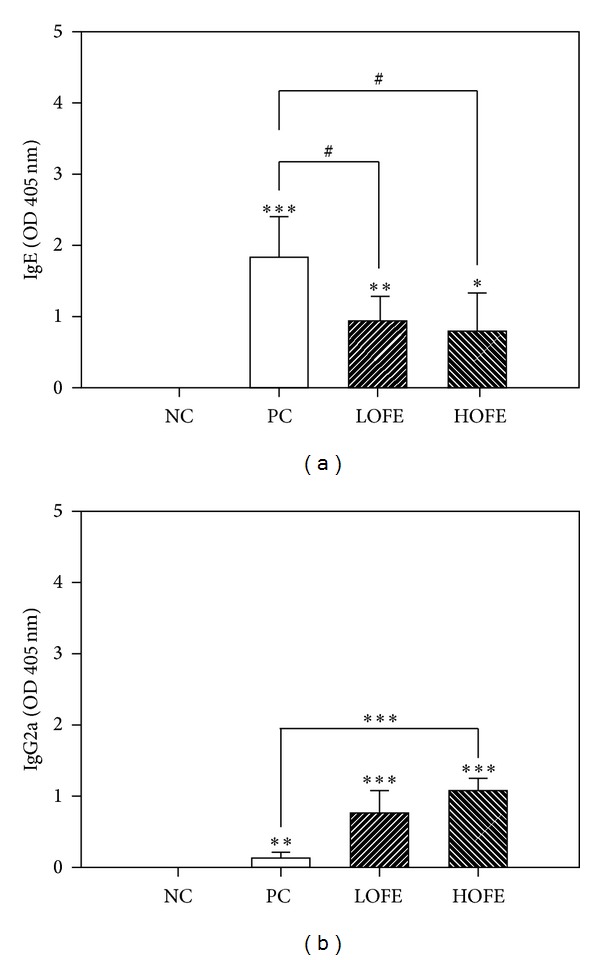
The effects of daily oral administration of OFE on the OVA-specific IgE and IgG2a antibody production in the OVA animal model of allergic airway inflammation. PC: OVA immunized group; NC: negative control group; HOFE: high dose of OFE; LOFE: low dose of OFE. Significant increase *P < 0.05,**P < 0.01, and ***P < 0.001 or decrease ^#^ *P* < 0.05, ^##^ *P* < 0.01, and ^###^ *P* < 0.001, as compared to NC or PC groups.

Following the OFE preventive protocol (Figure 1), OFE oral administration inhibited OVA-specific IgE production (Figure 2(a)) and enhanced the production of OVA-specific IgG2a (Figure 2(b)). IgE suppression can alleviate the development of asthma [47]. In contrast, IgG2a is the major isotype of the Th1 immune response. The effect of antioxidants to regulate the immune response has been an important issue in western society [48]. The strong Th2 inhibition by flavonoids [42] and phenolic compounds [49] contributes to the suppression of Th2 inflammation. The polarization effect of antioxidants on Th1 and Th2 has been discussed [50]. In a study of the antiallergic effects of curcumin, IFN-*γ* was increased and IgE was suppressed in the latex allergy model [51]. In our study, the increase in anti-OVA IgG2a and decrease in IgE expression indicate that OFE prevents development of Th2 suppression. A higher GSH induction also contributes to the development of Th1 as suggested by the Th1-promoting effect of GSH [24].

### 3.5. Cellular Composition in Bronchoalveolar Fluid in an Animal Model of Allergic Airway Inflammation and the Histopathologic Effects of OFE on Mouse Lung Tissue

**Figure 3:**
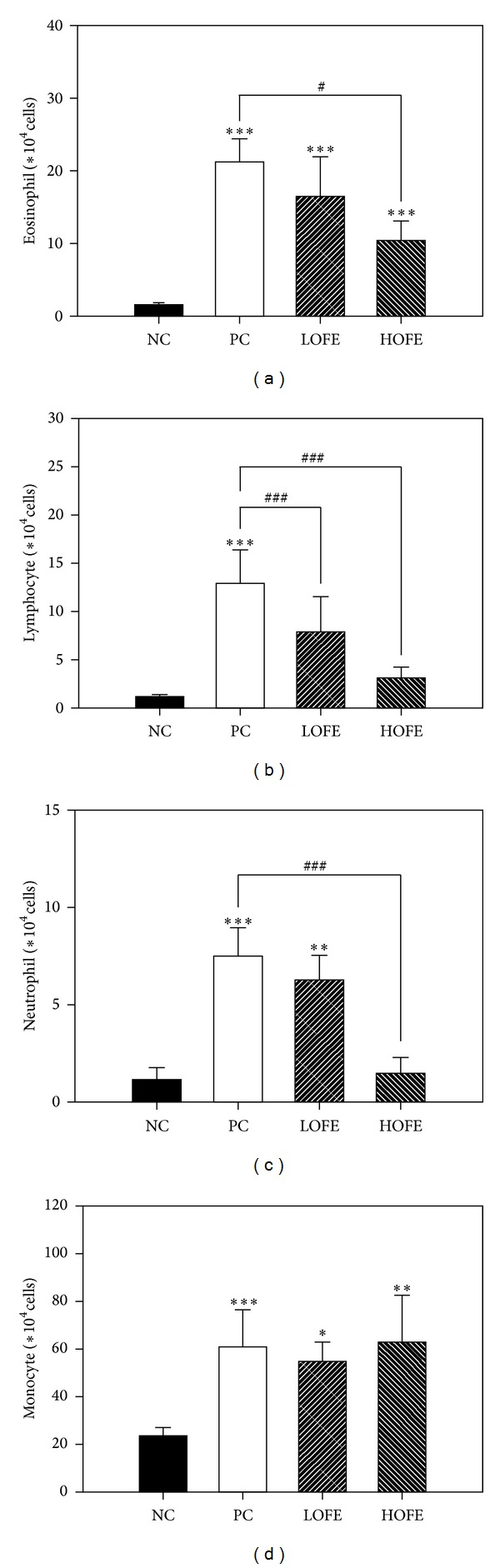
The effects of OFE on cell (eosinophils, neutrophils, monocytes, or lymphocytes) infiltration in bronchoalveolar fluid. Significant increase *P < 0.05, **P < 0.01, and ***P < 0.001 or decrease ^#^ *P* < 0.05, ^##^ *P* < 0.01, and ^###^ *P* < 0.001 as compared to NC or PC groups.

**Figure 4:**
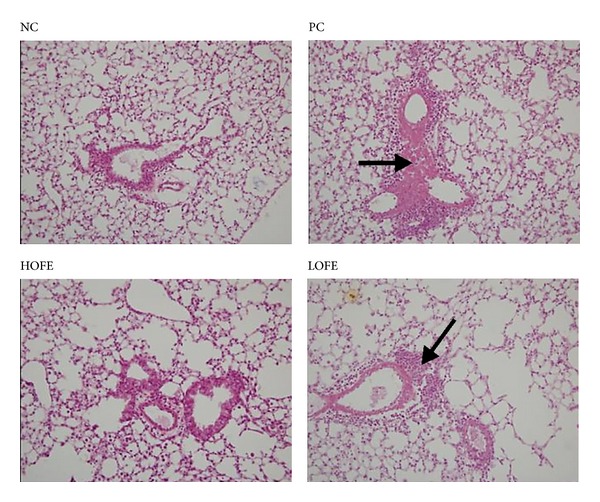
Lung sections were prepared from mice subjected to the OVA animal model of allergic airway inflammation after daily oral OFE or saline (control) administration. Arrows indicate immune cell infiltration by hematoxylin-eosin staining (magnification 200x).

In order to evaluate the effects of OFE on the recruitment of inflammatory cells into airway, cells in bronchoalveolar(BAL) fluid were determined by Liu's stain. Compared with the positive control group, oral administration of a high dose (HOFE) but not a low dose (LOFE) of OFE could suppress the recruitment of eosinophils, neutrophils, and lymphocytes (Figure 3). In the histopathological study, extensive cellular infiltration of the periairway region in lung sections of positive control-group mice was found. However, lung tissue from groups administered OFE demonstrated less severe inflammation (Figure 4). Fewer inflammatory cells infiltrated the periairway region in lungs of mice in the HOFE group. This result is consistent with the cellular composition of BAL fluid in the HOFE group and with the observed antiallergic effect of OFE. Results from previous studies examining the antiallergic effects of verbascoside [43] and rutin [45] demonstrated that these compounds in OFE contribute to the inhibition of inflammatory cell infiltration in lung.

### 3.6. Effects of Oral OFE Administration on Relative Organ Weight in an Animal Model of Allergic Airway Inflammation

A change in organ weights is a good indicator of chemically- or biologically-induced damage to organs [52]. Oral administration of OFE (100, 800, or 1000 mg/kg) for 28 days did not alter organ weights (liver, spleen, lung, and kidney) in mice (data not shown). However, HOFE (1000 mg/kg) administration for 28 days to mice subjected to the OVA animal model of allergic airway inflammation lowered the relative kidney weight to body weight percentage when compared with mice in the negative control group (NC group, treated with saline) but not when compared with mice in the positive control (PC group, OVA-immunized and treated with saline) (Table 2). In contrast, there were no significant differences among groups in the relative organ weight to body weight percentages for liver and lung. Recently, we evaluated a medium dose of OFE (500 mg/kg) in the OVA-induced allergic airway inflammation model and found that medium dose of OFE also protected against the infiltrated cells in BAL fluid and maintained the antioxidative state in lung (data not shown). Further investigation will be required to clarify the organ cytotoxicity effect by a medium dose of OFE orally administered in this animal model of allergic airway inflammation.

### 3.7. OFE Improves Antioxidative Status in Mouse Lung following OVA Administration

**Figure 5:**
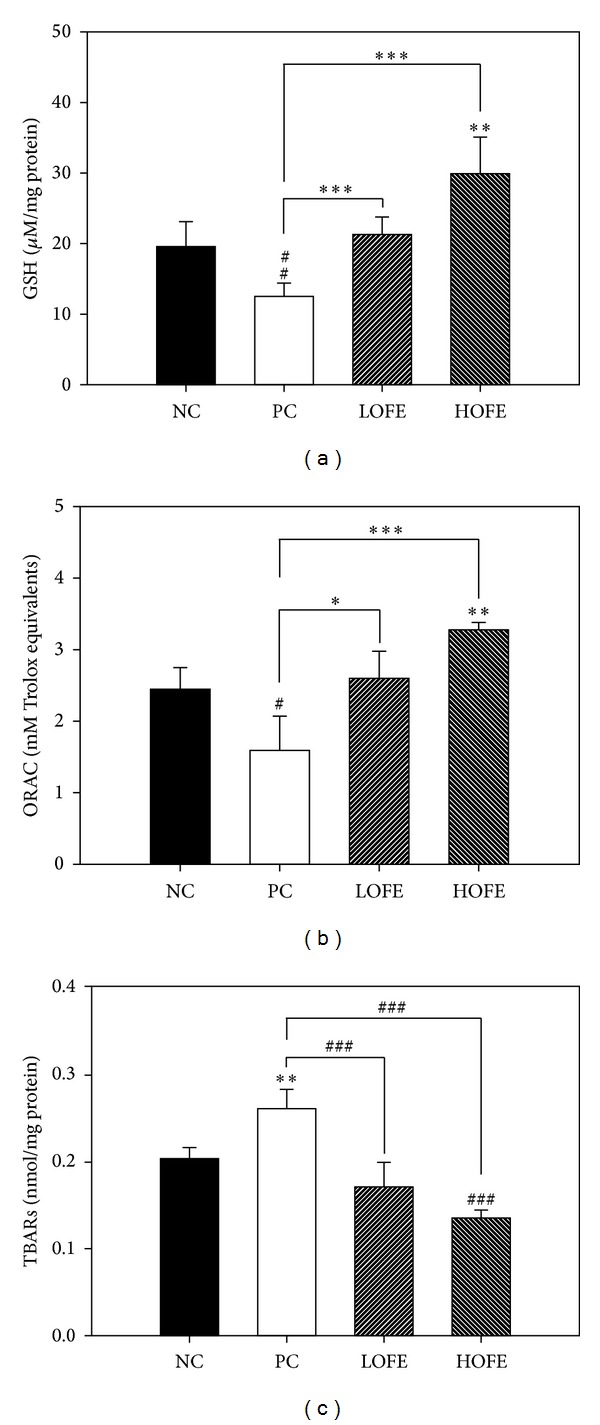


We measured the severity of the oxidative damage in lungs of mice from each group. In lungs taken from animals subjected to the mouse model of allergic airway inflammation, the GSH concentration and the total antioxidant capacity values obtained from the ORAC assay were significantly decreased compared with those of the lungs from mice in the negative control group. This suggests that airway inflammation may lead to oxidative stress in lungs. However, these phenomena were improved following oral administration of OFE (Figures 5(a) and 5(b)). In contrast, the value obtained in the TBARS assay for mice treated with OFE was significantly lower than that obtained for mice in the positive control group (*P* < 0.01, Figure 5(c)). Taken together, these data indicate that OFE improves the total antioxidant capacity in lung and that this improvement is correlated with a lower severity of signs in the OFE-treated mice subjected to the OVA-induced animal model of allergic airway inflammation. Recent reports have suggested that increasing the GSH levels could attenuate Th2 development [24–27], which offers a possible mechanism for the alleviation in the severity of airway inflammation observed in the present study following OFE administration.

## 4. Conclusion 

The therapeutic success of many antioxidant agents in allergic airway inflammation in the clinic is moderate at best [53, 54]. Conversely, researchers have found that antioxidants such as fruit juice [55] and plant extracts [56] interfere with the Th1 immune response in human peripheral blood mononuclear cells. Such results reflect the need for determining the appropriate antioxidants for treatment in different types of inflammatory disease. The present report was the first to evaluate the antioxidant and immunomodulatory effects of OFE, applying traditional Chinese medicinal knowledge regarding the *O. fragrans* flower to modern scientific study. We found that OFE, which contains many antioxidants, promotes a positive antioxidative state in an animal model of allergic airway inflammation. It also has protective effects including decreasing the OVA-specific IgE production and inflammatory cell infiltration in lung.

## Figures and Tables

**Table 1:** Effects of orally administered OFE on oxidative status in the lungs and spleen of mice as determined in three different assays.

Tissue	ORAC (Trolox equivalents, mM)		TBARS (nM/mg protein)		GSH (*μ*M/mg protein)	
	Control	OFE	Control	OFE	Control	OFE
Lung	2.42 ± 0.61	3.60 ± 0.24*	0.20 ± 0.02	0.12 ± 0.02*	15.27 ± 3.14	24.36 ± 3.01***
Spleen	2.10 ± 0.68	3.97 ± 0.79***	0.57 ± 0.01	0.46 ± 0.05***	15.97 ± 1.13	25.23 ± 1.19***

OFE (1000 mg OFE/kg body weight in 200 *μ*L normal saline) was orally administered daily for 14 days.

Data are expressed as mean ± standard deviation; *n* = 4; *P < 0.05, ***P < 0.01 as compared to (control) BALB/c mice given normal saline.

**Table 2:** Effects of OFE on relative organ weights for mice in an animal model of allergic airway inflammation.

Relative organ weight to body weight (%)				
Group				
Organ	NC	PC	LOFE	HOFE
Liver	6.00 ± 0.86	5.04 ± 0.36	5.34 ± 0.68	5.44 ± 0.80
Lung	1.38 ± 0.31	1.13 ± 0.14	1.33 ± 0.21	1.34 ± 0.17
Kidney	1.75 ± 0.20	1.48 ± 0.05	1.51 ± 0.14	1.42 ± 0.10^#^

Evaluation of OFE cytotoxicity in an animal model of allergic airway inflammation. Mice (*n* = 4) were injected (i.p.) with OVA for positive control (PC), a low dose of OFE (LOFE) or a high dose of HOFE. Negative control (NC) mice were injected with normal saline. During the establishment of the allergic airway inflammation model, mice were treated for 28 days with saline (NC and PC), 1000 mg OFE/kg body weight (HOFE, in 200 *μ*L saline), or 100 mg OFE/kg body weight (LOFE, in 200 *μ*L saline). After sacrifice on Day 40, organs were collected and weighed. Data are expressed as mean ± standard deviation. Significant decrease ^#^ *P* < 0.05 compared to NC group mice.
